# Karyotype analysis of amniotic fluid cells and report of chromosomal abnormalities in 15,401 cases of Iranian women

**DOI:** 10.1038/s41598-021-98928-3

**Published:** 2021-09-30

**Authors:** Sarang Younesi, Mohammad Mahdi Taheri Amin, Sedigheh Hantoushzadeh, Pourandokht Saadati, Soudabeh Jamali, Mohammad-Hossein Modarressi, Shahram Savad, Saeed Delshad, Saloomeh Amidi, Taraneh Geranorimi, Fariba Navidpour, Soudeh Ghafouri-Fard

**Affiliations:** 1 Prenatal Screening Department, Nilou Laboratory, Tehran, Iran; 2 Genome-Nilou Laboratory, Tehran, Iran; 3 Maternal Fetal and Neonatal Research Center, Valiasr Hospital, Tehran University of Medical Sciences, Tehran, Iran; 4 Clinical and Anatomical Pathologists, Prenatal Screening Department, Nilou Laboratory, Tehran, Iran; 5 Post Analytical Quality Control Department, Nilou Laboratory, Tehran, Iran; 6 Department of Medical Genetics, School of Medicine, Tehran University of Medical Sciences, Tehran, Iran; 7 Department of Medical Genetics, Genome-Nilou, Tehran, Iran; 8 Obstetrics and Gynecology Department, Tehran University of Medical Sciences, Imam Khomeini Hospital, Tehran, Iran; 9 Department of Medical Genetics, Shahid Beheshti University of Medical Sciences, Tehran, Iran

**Keywords:** Genetics, Structural biology, Diseases

## Abstract

The aim of present study was to assess the karyotypes of amniotic fluid cells and find the frequency of chromosomal abnormalities and their significance in clinical setting. A total of 15,401 pregnant women were assessed from March 2016 to May 2019, and 14,968 amniotic fluid samples were successfully cultured. These fetuses were grouped according to different indications including advanced maternal age, abnormal nuchal translucency (NT) values, positive first/second trimester screening results, high risk NIPT results, very low PAPP-A and free β-hCG multiples of the normal median (MoM) results, abnormal ultrasound findings or previous history of chromosomal abnormalities. Results indicated the presence of normal karyotype in 90.2% (13,497/14,968) of fetuses. Totally, 46.4% (6945/14,968) of fetuses were 46,XX and 43.8% (6552/14,968) had 46,XY chromosome pattern. A total of 1077 abnormal karyotypes were found among 14,968 fetuses, thus the rate of abnormal fetuses was calculated to be 7.2% (1072/14,968). Meanwhile, a total of 394 cases (2.8%) had a normal polymorphism in their karyotype. In other words, abnormal karyotypes were detected in one of 13.9 cases of patients underwent amniocentesis. Down syndrome, Edward’s syndrome, abnormal mosaicisms and Patau’s syndrome were detected in 4.4% (659/14,968), 0.57% (85/14,968), 0.49% (74/14,968) and 0.24% (36/14,968) of cases, respectively. Sex chromosomal abnormalities including Klinefelter syndrome, Turner syndrome and 47,XXX karyotype were detected in 64 cases (0.43%). In this article, the rates of chromosomal abnormalities are compared between different groups of patients based on the advanced maternal age, abnormal NT values, very low PAPP-A and free β-hCG MoMs results, and positive FTS results. The current investigation provides insight into the most appropriate indications for amniocentesis in Iran.

## Introduction

Chromosomal abnormalities are considered as an important cause of congenital defects and intellectual disability^1^. These abnormalities are not curable at present, and are associated with both financial and psychological burden. These disorders have a combined frequency of 1 in 153 births, necessitating conduction of screening programs for identification of affected fetuses at appropriate gestational age^2^. Measurement of biochemical markers in maternal circulation, ultrasonographic methods, and non-invasive prenatal diagnosis (NIPT) tests based on cell-free fetal DNA in maternal plasma have been proposed as efficient screening methods^2^. Moreover, Committee Opinion of ACOG and SMFM have reaffirmed their reviews in 2020 and made recommendations regarding the application of newer genetic technologies (such as chromosomal microarray—CMA) in the prenatal setting^3^. However, due to the limitation of access to a high resolution CMA, the karyotype method is still used as the best diagnostic method during pregnancy. The appropriate cut-off point for conduction of amniocentesis is a subject of debates nowadays. Karyotype analysis not only helps in the identification of chromosomal abnormalities in fetus, but also offers the scientific basis for either continuation or termination of pregnancy and reduction of the incidence of birth defects^4^. In the current study, we provide an overview of the detected chromosomal abnormalities in a large cohort of Iranian pregnant women underwent amniocentesis due to different indications including advanced maternal age, abnormal nuchal translucency (NT) values, positive first/second trimester screening (FTS/STS) results, high risk NIPT results, very low PAPP-A and free BhCG multiples of the normal median (MoM) result, abnormal ultrasound findings or previous history of chromosomal abnormalities. Then, we compare the frequencies of chromosomal abnormalities between different groups of patients based on the advanced maternal age, abnormal NT values, very low PAPP-A and free β-hCG Moms results and positive first FTS results.

## Methods

### Cases

In the present retrospective study, we collected the results of 15,401 amniocentesis samples sent for analysis to Genome-Nilou Medical Laboratory, Tehran, Iran. Amniocenteses were performed between March 2016 and May 2019. Detailed demographic and clinical data were collected through filling questionnaires, direct interview with pregnant women and assessment of medical records. The results of serum free β-hCG, PAPP-A, β-hCG, AFP, uE3 and inhibin A MoMs and other clinical and sonographic reports of patients were documented. The study protocol was approved by the ethical committee of Tehran University of Medical Science and the study protocol was performed in accordance with the relevant guidelines. Informed written consent forms were obtained from study participants.

Since Nilou laboratory is a referral center, in two third of cases serum markers, particularly STS and sequential tests were checked in other centers. Women underwent cytogenetic analyses after appropriate genetic counseling with complete explanation of methods, risks, and indications. Samples were collected by expert perinatologists or Maternal–Fetal medicine specialists through ultrasound-guided transabdominal puncture and the amniotic fluid samples were taken in two separate tubes and sent to the laboratory. Amniotic fluid cells of each tube were cultured in long-term cell cultures in Amniomax medium (Gibco, USA). Each medium was incubated separately at 37 °C in CO_2_ incubator (Memmert, Germany). Standard Giemsa banding was used for assessment of chromosomes. Chromosomal analyzes were performed simultaneously by an FDA approved software (Gen ASIS, Canada) and manually by an expert Cytogentist using an Invert Microscope (LABO Med, USA).

For about 58.5% of patients, QF-PCR (Dseyver, Sweden) test was requested at the same time. The main reason for requesting this test was gestational age ≥ 17 W + 0D, according to the time limit for legal termination of affected pregnancies in Iran which is 18 W + 6D.

### Statistical methods

Statistical analyses were performed using SPSS Statistics software version 21 (IBM Corporation, Armonk, NY, USA). Chi-2 test was used for assessment of association between categorical variables. The relationships between quantitative variables were evaluated using ANOVA test. *P* values < 0.05 were considered as significant. The Kruskal–Wallis test was used as a non-parametric test equivalent to ANOVA for comparing two or more independent samples with similar or different sample sizes. This test was used for comparison of MoM values of circulatory markers between pregnancies with normal and abnormal karyotypes.

## Results

A total of 15,401 amniocenteses were performed in the center. Karyotype analyses were not performed in 802 cases due to different reasons (Table 1).

**Table 1:** Reasons for lack of karyotype data in a proportion of performed amniocenteses.

Parameter	Valid no.	Percent (%)
Contamination with maternal blood	23	2.9
Culture failure	97	12.1
Request of only QF-PCR	279	34.8
Request for molecular tests for single gene disorders	162	20.2
Patients’ request for sample withdraw	27	3.4
Inadequate sample	73	9.1
Request for only array-CGH	141	17.5
Total	802	100

Detailed demographic and clinical data of cases are summarized in Table 2. A total of 6923 amniocentesis i.e., 44.9% of total cases were performed in cases aged more than 35 years. Eighty-two (0.5%) and 30 (0.2%) of cases had previous history of Down syndrome and other chromosomal abnormalities, respectively. In addition, 359 (2.4%) of pregnancies were twin pregnancies. Finally, 1098 (9.3%) of cases had NT > 3.0 mm. Regarding the consanguinity parameter, we had data of 1656 cases, 393 (23.7%) of them being consanguineous and 1263 (76.3%) having no consanguinity.

**Table 2:** Demographic and clinical data of patients (valid number column indicate the available data for each parameter).

Parameter	Valid no.	Mean	SD	Minimum	Maximum
Age (years)	15,401	33.3	5.8	14	52
Age 35–39	4812 (32.1%)				
Age > 39	2111 (14.1%)				
Gestation age (week +day)	1628	17 W + 2D	2 W + 1D	13 W + 1D	28 W + 4D
Weight	1294	69.9	12.4	41	99

Results of a total of 14,968 karyotypes were reported (Table 3). Results indicated the presence of normal karyotype in 90.2% (13,497/14,968) with 46.4% (6945/14,968) having 46,XX and 43.8% (6552/14,968) having 46,XY chromosome pattern. Total of 1077 abnormal karyotypes were found among 14,968 fetuses, thus the rate of abnormal fetuses was calculated to be 7.2% (1072/14,968). Meanwhile, a total of 394 cases (2.6%) had a normal polymorphic karyotype. In other words, abnormal karyotypes were detected in one per 13.9 cases of patients underwent amniocentesis.

**Table 3:** Frequencies of different normal, abnormal and polymorphic karyotypes in fetuses (OAPR: Odds of being affected given a positive result).

Karyotype	Frequency	Valid percent	OAPR
46,XY	6552	43.8	
46,XX	6945	46.4	
Total of normal results	13,497	90.2	
**Abnormal chromosomal abnormalities**			
Down syndrome	659	4.4	1:22.7
Edward’s syndrome	85	0.57	
Abnormal mosaicisms	74	0.49	
Patau’s syndrome	36	0.24	
Klinefelter syndrome	25	0.17	
Turner	22	0.15	
47,XXX (super female)	17	0.11	
69,XXY or XXX	5	0.03	
Other chromosomal abnormalities	154	1.0	
Total of chromosomal abnormalities	1077	7.2	1:13.9
**Normal chromosomal polymorphisms**			
46,XX or XY, inv(9) (p11; q12)	131	0.87	
46,XY or XX,15 ps+	51	0.34	
46,XY,qh-	36	0.24	
46,XY or XX,1qh+	25	0.17	
46,XY or XX,21 ps+	17	0.11	
46,XY or XX,22 ps+	13	0.09	
Other normal chromosomal polymorphisms	121	0.82	
Total of normal chromosomal polymorphisms	394	2.6	1:37.9
Total	14,968	100	

Down syndrome, Edward’s syndrome, abnormal mosaicisms and Patau’s syndrome were detected in 4.4% (659/14,968), 0.57% (85/14,968), 0.49% (74/14,968) and 0.24% (36/14,968) of cases, respectively. The rate of male fetuses with Down syndrome to female fetuses with Down syndrome has been calculated to be 54%/45.6%, indicating higher rates of this abnormality among male fetuses (*P* < 0.001). Normal polymorphisms were detected in 2.6% of the assessed fetuses with 46,XX or XY, inv(9) (p11; q12) being the most frequent polymorphism. Sex chromosomal abnormalities including Klinefelter syndrome, Turner syndrome, 47,XXX karyotypes were detected in 64 cases (0.43%).

Then, we assessed the frequency of normal polymorphisms, Down syndrome and other chromosomal abnormalities based on the defined indications for amniocentesis (Table 4). At least, 13.0% of cases had two or more indications for amniocentesis. Advanced maternal age (≥ 35 years) was the only criterion for indicating amniocentesis in 11.3% of cases. The best Odds of being affected given a positive result (OAPR) was calculated for free βHCG < 0.2 MoM, i.e. one chromosomal abnormality per 1.1 amniocentesis. After this indication, three of the top OAPR values for amniocentesis indications were high risk NIPT (one chromosomal abnormality per 1.4 amniocentesis), PAPP-A MoM < 0.26 (one chromosomal abnormality per 1.7 amniocenteses) and Free BhCG MoM > 5.0 (one chromosomal abnormality per 2 amniocenteses). On the other hand, the poorest OAPR was obtained for intermediate risk for Trisomy 21 used for FTS (one chromosomal abnormality per 52.2 amniocenteses).

**Table 4:** Frequency of normal polymorphisms, Down syndrome and other chromosomal abnormalities based on the defined indication for amniocentesis (MoM: multiple of the median, NIPT: non-invasive prenatal testing).

Parameter	Sub group	Valid no.	Percent (%)	Normal polymorphisms	Down Syndrome	Other chromosomal abnormalities	OAPR (Down syndrome + Other chromosomal abnormalities)
Age > 35	35–39	940	6.3	19	14	18	1:29.3
	≥ 40	576	5.0	17	7	13	1:28.8
NT > 3 mm	3.0–3.4	533	3.6	15	60	11	1:6.9
	≥ 3.5	556	3.7	8	124	47	1:3.2
Positive first trimester screening results	T21	5131	34.3	136	315	118	1:11.8
	T18	351	2.3	4	53	28	1:4.3
	T13	441	2.9	11	83	40	1.3.6
Positive second trimester screening results	T21	3971	26.5	79	53	48	1:39.3
	T18	114	0.76	1	1	4	1:22.8
	SLOS	29	0.2	0	0	0	–
	NTDs	59	0.4	4	1	4	1:11.8
Positive sequential screening results	T21	769	5.1	18	13	10	1:33.4
	T18	14	0.09	1	0	1	1:14
	T13	113	0.75	5	16	13	1:3.9
Only Intermediate Risk	T21	1304	8.7	44	9	16	1:52.2
	T18	38	0.25	0	0	0	–
	T13	36	0.24	1	0	0	–
PAPP-A MoM < 0.26		112	0.75	0	40	26	1:1.7
Free βHCG MoM < 0.2		18	0.12	0	4	12	1:1.1
Free βHCG MoM > 5.0		63	0.42	0	28	3	1:2
Second trimester abnormality in ultrasound (including soft markers)		1076	7.2	26	58	53	1:9.7
NIPT Positive		515	3.4	5	295	59	1:1.4
NIPT low fetal fraction	< 4.0%	39	0.26	5	2	4	1:6.5
Previous history of Down Syndrome		82	0.55	1	2	2	1:20.5
Previous history of other chromosomal abnormalities		30	0.2	1	1	6	1:4.3
Total	14,968	16,910	113.0				

Then, we assessed the association between chromosomal abnormalities and maternal age (Table 5). Results of Chi-2 test showed that frequency of chromosomal abnormalities, including Down syndrome increases with increasing maternal age (*P* < 0.001). Higher and lower frequencies of chromosomal abnormalities were detected in 35–39 and < 20 years age groups, respectively. Moreover, the highest frequencies of chromosomal abnormalities (excluding Down syndrome) and normal polymorphisms were reported among patients less than 20 years old which is possibly due to the fact that in this age group only patients with abnormal screening results underwent amniocentesis. Finally, Chi-2 tests showed significant increase in chromosomal abnormalities parallel with increase in maternal age from 25 years (*P* < 0.001). However, the frequency of normal polymorphisms was not associated with maternal age.

**Table 5:** Association between maternal age and presence of chromosomal abnormalities.

		Karyotypes					Total
		46,XY	46,XX	Normal polymorphisms	Down syndrome	Other abnormalities	
**Age groups**							
< 20	Count	82	66	7	5	9	169
	% within age group	48.5%	39.1%	4.1%	3.0%	5.3%	1.13%
20–24	Count	526	486	24	33	27	1096
	% within age group	48.0%	44.3%	2.2%	3.0%	2.5%	7.33%
25–29	Count	1527	1534	75	118	72	3326
	% within age group	45.9%	46.1%	2.3%	3.5%	2.2%	22.24%
30–34	Count	1498	1626	110	131	76	3441
	% within age group	43.5%	47.3%	3.2%	3.8%	2.2%	23.01%
35–39	Count	2020	2298	117	252	125	4812
	% within age group	42.0%	47.8%	2.4%	5.2%	2.6%	32.18%
> 39	Count	896	928	61	154	72	2111
	% within age group	42.4%	44.0%	2.9%	7.3%	3.4%	14.11
Total	Count	6549	6938	394	693	381	14,955
	% within age group	43.8%	46.4%	2.6%	4.6%	2.5%	100.0%

Subsequently, we assessed association between the frequency of chromosomal abnormalities and maternal weight as well as gestational age (Table 6).

**Table 6:** Association between the frequency of chromosomal abnormalities and maternal weight as well as gestational age.

Karyotypes	Gestational age (days)	Maternal weight
**46,XY**		
Mean	122.43	69.03
N	672	418
SD	16.155	11.907
**46,XX**		
Mean	122.75	70.15
N	798	408
SD	15.499	11.970
**Normal polymorphisms**		
Mean	119.53	76.05
N	38	22
SD	13.190	13.326
**Down syndrome**		
Mean	108.84	69.31
N	43	323
SD	7.653	12.600
**Other chromosomal abnormalities**		
Mean	113.20	71.65
N	49	106
SD	20.111	13.098

ANOVA test showed that mean maternal weight was higher in those having normal polymorphisms (*P* = 0.001). Therefore, the frequency of normal polymorphisms was higher in obese patients. Yet, there was no association between chromosomal abnormalities and maternal weight.

The frequency of chromosomal abnormalities was higher in non-consanguineous marriages compared with consanguineous ones (*P* < 0.001). Frequency of Down syndrome in the former group was 33.8%, while in the latter group was 24.4%. Other chromosomal abnormalities were detected in 8.7% and 10.6% of consanguineous and non-consanguineous marriages, respectively. Table 7 shows the results of this type of comparison.

**Table 7:** Distribution of different karyotypes among consanguineous and non-consanguineous marriages.

		Karyotypes					Total
		46,XY	46,XX	Normal polymorphisms	Down syndrome	Other abnormalities	
**Relation**							
Consanguineous	Count	129	131	3	96	34	393
	% within group	32.8%	33.3%	0.8%	24.4%	8.7%	100.0%
Non-consanguineous	Count	357	324	21	427	134	1263
	% within group	28.3%	25.7%	1.7%	33.8%	10.6%	100.0%
Total	Count	486	455	24	523	168	1656
	% within group	29.3%	27.5%	1.4%	31.6%	10.1%	100.0%

We also assessed association between NT value and frequency of chromosomal abnormalities (Table 8).

**Table 8:** Association between NT value and frequency of chromosomal abnormalities.

		Karyotypes					Total	OAPR (Down syndrome + other chromosomal abnormalities)
		46,XY	46,XX	Normal polymorphisms	Down syndrome	Other abnormalities		
**NT**								
< 3	Count	4579	4968	293	416	232	10,488	1:16.1
	% within group	90.1%	94.0%	92.7%	69.3%	80.0%	90.6%	
3–3.5	Count	281	166	15	60	11	533	1:6.9
	% within group	5.5%	3.1%	4.7%	10.0%	3.8%	4.6%	
> 3.5	Count	224	153	8	124	47	556	1:3.2
	% within group	4.4%	2.9%	2.5%	20.7%	16.2%	4.8%	
Total	Count	5084	5287	316	600	290	11,577	1:13.0
	% within group	100.0%	100.0%	100.0%	100.0%	100.0%	100.0%	

In brief, 9.4% of fetuses had NT values greater than 3 mm. Notably, 4.8% of fetuses had NT values more than 3.5 mm. Chi-2 test showed higher prevalence of chromosomal abnormalities in fetuses with NT values higher than 3 or 3.5 mm (*P* < 0.001). In fact, 92% of fetuses with normal karyotypes, 92.7% of fetuses with normal polymorphisms, 69.3% of Down syndrome fetuses and 80% of fetuses with other chromosomal abnormalities had NT values < 3 mm. On the other hand, 3.7% of fetuses with normal karyotypes, 2.6% of fetuses with normal polymorphisms, 20.7% of Down syndrome fetuses and 16.2% of fetuses with other chromosomal abnormalities had NT values more than 3.5 mm. It is worth mentioning that although the frequency of chromosomal abnormalities increases when NT increases, the majority of cases with chromosomal abnormalities are detected in the fetuses with NT < 3 mm, because the number of patients in this group is approximately 9.6 times that of patients with an NT greater than 3 mm. Therefore, one per 6.9 fetuses with NT > 3 mm, one per 3.2 fetuses with NT > 3.5 and one per 16.1 fetuses with NT < 3 mm would be diagnosed as having Down syndrome and other chromosomal abnormalities. If we set the threshold at NT value of 3 mm (instead of 3.5 mm), we could detect 60 (10%) additional cases of Down syndrome and 11 (3.8%) additional cases of other chromosomal abnormalities. However, when the threshold is set at NT > 3.5 mm, referral for amniocentesis decreases from 9.4 to 4.8%. With the advent of NIPT, we can simultaneously lower the amniocentesis rate (by applying cut-off point of 3.5 mm for NT) and detecting additional cases of chromosomal abnormalities, particularly Down syndrome cases.

Then, we assessed association between free β-hCG and PAPP-A levels and chromosomal abnormalities. When all kinds of chromosomal abnormalities are combined, one per 1.7 pregnancies with PAPP-A levels < 0.26 will be diagnosed as affected. On the other hand, when all kinds of chromosomal abnormalities are combined, one per 1.1 pregnancies with free β-hCG levels < 0.2 MoM will be diagnosed as affected.

Moreover, frequency of chromosomal abnormalities was higher in pregnancies with free β-hCG levels > 5 MoM (*P* = 0.01). One per 1.3 pregnancies that underwent amniocentesis due to free β-hCG levels > 5 MoM, one was diagnosed as Down syndrome or other chromosomal abnormalities. Therefore, free β-hCG levels > 5 MoM is an important indicator for chromosomal abnormalities (Table 9).

**Table 9:** Association between PAPP-A and free β-hCG levels and chromosomal abnormalities.

		Karyotypes					Total
		46,XY	46,XX	Normal polymorphisms	Down syndrome	Other abnormalities	
**PAPP-A**							
< 0.26 MoM	Count	29	17	0	40	26	112
	% within group	25.9%	15.2%	0.0%	35.7%	23.2%	100.0%
> 0.26 MoM	Count	341	337	21	266	71	1036
	% within group	32.9%	32.5%	2.0%	25.7%	6.9%	100.0%
Total	Count	370	354	21	306	97	1148
	% within group	32.2%	30.8%	1.8%	26.7%	8.4%	100.0%
**Free β-hCG**							
< 5 MoM	Count	364	342	21	279	94	1100
	% within group	33.1%	31.1%	1.9%	25.4%	8.5%	100.0%
> 5 MoM	Count	16	16	0	28	3	63
	% within group	25.4%	25.4%	0.0%	44.4%	4.8%	100.0%
Total	Count	380	358	21	307	97	1163
	% within group	32.7%	30.8%	1.8%	26.4%	8.3%	100.0%
< 0.2 MoM	Count	1	1	0	4	12	18
	% within group	0.3%	0.3%	0.0%	1.3%	12.4%	1.5%
> 0.2 MoM	Count	379	357	21	303	85	1145
	% within group	99.7%	99.7%	100.0%	98.7%	87.6%	98.5%
Total	Count	380	358	21	307	97	1163
	% within group	100.0%	100.0%	100.0%	100.0%	100.0%	100.0%

In FTS for Down syndrome, 30.2% of Down syndrome cases, 4.5% of Edward’s syndrome cases, 13.6% of Patau’s syndrome cases and 8.8% of other chromosomal abnormalities were in risk group of 1:10 or greater. Meanwhile, among 483 pregnancies within this group, 70.6% had normal karyotypes and 2.7% had normal polymorphisms. The frequencies of Down syndrome, Edward’s syndrome, Patau’s syndrome and other chromosomal abnormalities were 23.6%, 0.2%, 0.6% and 2.3%, respectively.

On the other hand, 83.5% of Down syndrome cases, 63.6% of Edward’s syndrome cases, 63.6% of Patau’s syndrome cases and 72% of other chromosomal abnormalities were in risk group of 1:250 or greater. Among 5131 pregnancies within this group, 88.9% had normal karyotypes and 2.6% had normal polymorphisms. The frequencies of Down syndrome, Edward’s syndrome, Patau’s syndrome and other chromosomal abnormalities were 6.1%, 0.3%, 0.3% and 1.8%, respectively. In other words, one per 11.8 pregnancies within this group had a pathologic chromosomal abnormality. Moreover, 14.9% of patients with Down syndrome were within the risk group of 1: 251 to 1: 1500, and 1.6% of patients with Down syndrome had a risk less than 1: 1500. From the total of the 1955 patients in this group, 1304 had no other reason for requesting amniocentesis, of which 9 patients (2.4%) had Down syndrome and 16 patients (9.5%) had other chromosomal abnormalities. In other words, from 52.2 patients referred for amniocentesis just for intermediate risk for Trisomy 21, only one case of chromosomal abnormality was diagnosed (Table 10).

**Table 10:** Distribution of chromosomal abnormalities within different groups based on the risk calculated in first trimester screening (FTS) for Down syndrome.

		Karyotypes						Total
		Normal	Normal polymorphisms	Down syndrome	Edward’s syndrome	Patau’s syndrome	Other abnormalities	
**FTS T21**								
< 10	Count	341	13	114	1	3	11	483
	% within group	5.1%	6.4%	30.2%	4.5%	13.6%	8.8%	6.5%
11–50	Count	991	30	90	2	1	24	1138
	% within group	14.9%	14.9%	23.9%	9.1%	4.5%	19.2%	15.4%
51–250	Count	3230	93	111	11	10	55	3510
	% within group	48.6%	46.0%	29.4%	50.0%	45.5%	44.0%	47.5%
251–1500	Count	1794	60	56	8	7	30	1955
	% within group	27.0%	29.7%	14.9%	36.4%	31.8%	24.0%	26.4%
> 1500	Count	289	6	6	0	1	5	307
	% within group	4.3%	3.0%	1.6%	0.0%	4.5%	4.0%	4.2%
Total	Count	6645	202	377	22	22	125	7393
	% within group	100.0%	100.0%	100.0%	100.0%	100.0%	100.0%	100.0%

The prevalence of trisomies 13 and 18 with risk scores up to 1:250 cut-off is not high. With cut-off point of 1:250 for these trisomies, it is possible to detect 86.5% of Down Syndrome cases, 100% of Edward’s syndrome cases, 92.9% of Patau’s syndrome cases and 70.6% of other chromosomal abnormalities. In other words, from 5.3 patients referred for amniocentesis just for this indication, only one case of chromosomal abnormality was diagnosed.

With the cut-off point of 1:250 for trisomy 18, it was possible to detect 91.7% of Down Syndrome cases, 100% of Edward syndrome cases, 75.0% of Patau’s syndrome cases and 77.8% of other chromosomal abnormalities. In other words, from 4.3 patients referred for amniocentesis just for this indication, only one case of chromosomal abnormality was diagnosed (Table 11).

**Table 11:** Distribution of chromosomal abnormalities within different groups based on the risk calculated in first trimester screening (FTS) for Edward and Patau syndromes.

		Karyotypes						Total
		Normal	Normal polymorphisms	Down syndrome	Edward’s syndrome	Patau ‘s syndrome	Other abnormalities	
**FTS T13**								
< 10	Count	50	1	27	7	2	2	89
	% within group	10.2%	7.1%	28.1%	46.7%	14.3%	11.8%	13.8%
11–50	Count	106	3	18	4	6	1	138
	% within group	21.6%	21.4%	18.8%	26.7%	42.9%	5.9%	21.3%
51–250	Count	151	7	38	4	5	9	214
	% within group	30.8%	50.0%	39.6%	26.7%	35.7%	52.9%	33.1%
251–1500	Count	133	3	11	0	1	3	151
	% within group	27.1%	21.4%	11.5%	0.0%	7.1%	17.6%	23.3%
> 1500	Count	51	0	2	0	0	2	55
	% within group	10.4%	0.0%	2.1%	0.0%	0.0%	11.8%	8.5%
Total	Count	491	14	96	15	14	17	647
	% within group	100.0%	100.0%	100.0%	100.0%	100.0%	100.0%	100.0%
**FTS T18**								
< 10	Count	47	0	20	9	2	1	79
	% within group	11.6%	0.0%	34.5%	60.0%	25.0%	11.1%	15.7%
11–50	Count	80	3	10	3	1	0	97
	% within group	19.8%	37.5%	17.2%	20.0%	12.5%	0.0%	19.3%
51–250	Count	139	1	23	3	3	6	175
	% within group	34.3%	12.5%	39.7%	20.0%	37.5%	66.7%	34.8%
251–1500	Count	108	4	5	0	2	2	121
	% within group	26.7%	50.0%	8.6%	0.0%	25.0%	22.2%	24.1%
> 1500	Count	31	0	0	0	0	0	31
	% within group	7.7%	0.0%	0.0%	0.0%	0.0%	0.0%	6.2%
Total	Count	405	8	58	15	8	9	503
	% within group	100.0%	100.0%	100.0%	100.0%	100.0%	100.0%	100.0%

## Discussion

In the current study, we provided an overview of the cytogenetic analyses in a large cohort of Iranian pregnant women referred for amniocentesis due to different indications, particularly advanced maternal age and positive FTS results. Based on the defined indications for amniocentesis, the best OAPR was calculated for free βHCG < 0.2 MoM, i.e. one chromosomal abnormality per 1.1 amniocenteses. High risk NIPT, PAPP-A MoM < 0.26, free BhCG MoM > 5.0 were the other amniocentesis indications with appropriate OAPR values. We detected abnormal karyotypes in one per 13.9 cases of patients underwent amniocentesis (1077/14,968 or 7.2%). Down syndrome, Edward’s syndrome, abnormal mosaicisms and Patau’s syndrome, sex chromosomal abnormalities (including Klinefelter syndrome, Turner syndrome, 47,XXX karyotypes) and normal polymorphisms were detected in 4.4%, 0.57%, 0.49% , 0.24%, 0.43% and 2.8% of cases, respectively. This finding is not concordant with Zhang et al. study in Shanghai, China^5^. In the study conducted by Zhang et al., a total of 388 abnormal karyotypes were found among 13,795 fetuses, and the rate of chromosomal abnormalities was 2.813% (388/13,795) or one per 35.6 cases. The main indications of amniocentesis in this study were high-risk serum screening, paternal/maternal abnormality, abnormal signs of ultrasound screening and advanced maternal age. Aneuploidies, autosomal structural abnormalities and mosaicisms were the most common identified karyotypes with frequencies of 59.8% (232/388), 24.7% (96/388) and 12.4% (48/388), respectively. Other uncommon abnormal karyotypes included marker chromosome (5/388, 1.3%), sex chromosomal structural abnormality (4/388, 1.0%) and triploidy (3/388, 0.8%)^5^. Our study is concordant with Li et al. study^6^ where among 4191 amniotic fluid specimens from Linyi, China in 2016–2017, a total of 358 abnormal karyotypes were detected, delineating the rate of 8.54% (358/4191 or one per 11.7 cases). Advanced maternal age and abnormal serological screening results were the major prenatal indications for pregnant women with chromosomal abnormalities. Autosomal aneuploidy was the most common pattern accounting for 64.53% of cases, of which 173 (48.32%) cases were trisomy 21, which was the main type of abnormal karyotypes, followed by trisomy 18 (14.25%). There were 38 cases with sex chromosome aneuploidies, including 47,XXY, 47,XXX, 47,XYY and 45,X accounting for 10.61% of the total chromosome abnormalities^6^. Our study is comparable with Xiao et al. study^7^. Among 12,365 pregnant women in their study, foetal abnormal karyotype was found in 428 (3.46% or one per 28.9). The detection rates of abnormal karyotype were 57.4% (1 per 1.7) in either a mother or father with chromosomal abnormality, 8.5% in the pregnant women with pathological ultrasound finding (1 per 11.8), 2.79% (1 per 35.8) in the pregnant women with advanced age (35 years and over) and 2.23% (1 per 44.8) in the women with abnormal maternal serum screening (MSS) tests^7^.

This study is also comparable with Wang et al.^4^ study in Changsha, China, 2013. They found that positive screening, advanced maternal age, and ultrasonography abnormality were the top 3 indications of amniocentesis and cordocentesis. They found 25 abnormal karyotypes in a total of 669 patients, including 6 cases of trisomy 21, 4 sex chromosomal abnormalities, 7 autosomal balanced translocations, 1 marker chromosome, and 7 mosaics^4^. This study is also comparable with Kessler et al.^8^ study in Port Alerge, Brazil, 2008. Among 879 cases, they detected 74 abnormal karyotypes (8.4%), the majority of them being found when the prior indication was fetal malformation^8^. Moreover, Caron et al.^9^ have shown that the overall frequency of chromosomal abnormalities for advanced maternal age (≥ 35 years) is 1.79%. In this group, 21% of all abnormalities were structural rearrangements (including markers) and less than half of all abnormalities were trisomy 21^9^.

The most important findings of the current study are identification of frequency of chromosomal abnormalities in each risk group based on the results of FTS. In FTS, we have demonstrated that with cut-off point of 1:250 for Trisomy 21, Trisomy 18 and Trisomy 13, the detection rates (DRs) were 79.1% (432/546), 90% (81/90) and 86.6% (123/142) for all abnormal chromosomal patterns. In other words, the poorest OAPR was obtained for intermediate risk for trisomy 21 used for FTS (one per 52.2 cases), so it seems that considering the intermediate risk in the first trimester screening is not remarkably effective and it is better to consider other follow up strategies (for example sequential or NIPT screening instead of amniocentesis) for this group to achieve 18.5% (101/546) improvement in DR. Huang et al. have shown in the risk group of 1:200 or greater, they could detect 222 of Down syndrome cases from 253 total cases (DR = 88%). They have suggested conduction of NIPT for the intermediate risk group (1:201–1:1500). This approach has led to detection of 18 additional cases of Down syndrome, 7% improvement in DR and 16% increase in false positive rate of the screening protocol^9^. This finding is concordant with Kagan et al. study^10^. They have shown that in the risk group between 1:101 and 1:1500, conduction of 3.6% extra procedures would lead to 7.2% improvement in DR^10^. Therefore, it is rational to suggest additional diagnostic tests or NIPT in women with defined risks between 1:251 and 1:1500^10^. Lindquist et al. have reported the highest frequency of chromosomal abnormalities in pregnancies with an FTS risk of more than 1:10 or MoM of serum markers less than 0.2. In fact, about half of pregnancies with MoM of serum markers less than 0.2 had risk values higher than 1:10^11^. Vogel et al. have conducted a retrospective study of 575 pregnancies underwent invasive testing due to an FTS risk higher than 1:300. They detected 22 cases of trisomies 21, 18 and 13, 14 cases of other aneuploidies and 15 cases with a pathogenic or probably pathogenic copy number variant (CNV). They have shown that with cut-off point of 1:150 in Contingent NIPT/invasive test, 100% of pathogenic CNVs can be detected. Authors have suggested application of cut-off point of 1:300 and PAPP-A MoM ≤ 0.2 for suggestion of amniocentesis and request for CMA. This study has also reported association between high NT and pathogenic CNVs and the presence of the majority of trisomies in the risk group of 1:50 and greater^12^.

Although the above mentioned studies might indicate that FTS can be supplemented by NIPT, sonograms, and possibly sequential screening, the model proposed in the current study can be applied in situations where NIPT/ultrasound is of limited availability.

The second important finding in the current study is that the frequency of other chromosomal abnormalities rather than Down Syndrome in patients with free βHCG < 0.2 MoM and PAPP-A MoM < 0.26 are 1–3 and 3–2, respectively. Our study is concordant with Xiao et al. study^7^. They showed in the pregnant women with abnormal MSS, 111 foetuses had abnormal karyotype, but only 36 foetuses had trisomy 13, 18 or 21. They have concluded that ultrasound is an important approach to prevent the birth of foetuses with chromosomal disease. Moreover, they have stated that non-invasive prenatal DNA detection cannot completely replace invasive prenatal diagnosis and MSS. Finally, they have proposed genetic amniocentesis for the pregnant women with certain indications^7^. Therefore, because in these cases there is a possibility of atypical chromosomal abnormalities (any chromosomal abnormality rather than trisomise 21, 18 and 13, monosomy X and sex chromosome trisomies such as XXX, XXY, XYY that are not detectable in the NIPT test), we strongly recommended amniocentesis instead of NIPT in two of above-mentioned indications.

Two other findings in recent study are remarkably interesting. First, the rate of male fetuses with Down syndrome to female fetuses with Down syndrome has been calculated to be 54%/45.6% (the sex ratio is 1.18), indicating higher rates of this abnormality among male fetuses. This is an important finding that although amniocentesis requests are higher in girls than in boys, the detected chromosomal abnormalities are higher in boys than in girls, which may mean that screening protocols are far more effective in boys than girls. In other words, male fetuses are more frequently affected with Down syndrome compared with female fetuses in spite of higher rate of positive screening results in female fetuses. This study is comparable with Vermra et al. study^13^. In this study, among total 75 children with trisomy 21, there were 42 males and 33 females. The sex ratio was 1.30 which was not statistically significant (*P* > 0.05). However, a similar sex ratio (1.36) was reported in a larger sample size which was statistically significant (*P* < 0.01) ^13^.

The association between abnormal βHCG and PAPP-A levels and abnormal findings other than trisomy 21 has also been reported in other studies. Thus, the present study has verified this finding in our population. The same is applicable for the higher male/female ratio of trisomy 21.

It is worth mentioning that for other chromosomal abnormalities rather than trisomy 21, cut-off points of 1:10 and 1:11–1:250 are not significantly different. Thus, it is not possible to define a cut-off point for high risk of these chromosomal abnormalities.

Moreover, the presence of chromosomal abnormalities was higher in non-consanguineous marriages compared with consanguineous ones. This is probably because the most common cause of miscarriage under 12 weeks is genetic disorders, which can be more in this group of patients, and as a result, children with problems have been aborted earlier than 12 weeks. Thus, consanguinity may remain an indication for FTS although not as the indication for amniocentesis.

Taken together, in the current study, we have presented the results of amniotic cells karyotypes in a large cohort of Iranian women underwent amniocentesis due to different indications and compared the results within these groups. We have also proposed three additional indications i.e. high risk NIPT, PAPP-A MoM < 0.26 and free BhCG MoM > 5.0 as the most important indications with appropriate OAPR values. Two latter indications have not been previously reported as indicators of invasive tests with high OAPR values. Thus, the present finding is an important novel finding of the current study. We have also shown that intermediate risk in FTS is not an appropriate indication for amniocentesis. Thus, elimination of this indication can improve the efficacy of screening protocols and reduce the number of unnecessary requests for amniocentesis.
